# Navigating Challenges in Sexual and Gender Minority Aging Research and Education: Advocacy and Community Action

**DOI:** 10.1093/geroni/igaf122.691

**Published:** 2025-12-31

**Authors:** Jace Flatt, Ethan Cicero

**Affiliations:** University of Nevada Las Vegas, Nevada, United States; Emory University, Atlanta, Georgia, United States

## Abstract

On October 16, 2016, the directors of the National Institute of Minority Health Disparities, with support from the Agency for Healthcare Research and Quality, designated sexual and gender minorities (SGMs)—including lesbian, gay, bisexual, transgender, queer, intersex, asexual, and additional identities (LGBTQIA+)—as a health disparity population for National Institutes of Health research. As of January 20, 2025, Executive Order 14168, titled “Defending Women from Gender Ideology Extremism and Restoring Biological Truth to the Federal Government,” has been used to overrule this designation and terminate federally-funded research involving SGM aging research, as well as rescind funding decisions and peer review of federal research grants including SGM aging populations. In this talk, we will highlight the current state of the science and challenges and pathways forward for education, training, and activism to support LGBTQIA+ aging populations and SGM aging research. This includes supporting early-career scholars, collecting sexual orientation and gender identity data in aging research, and developing strategies for advocating for terminated and rescinded research grants. We will provide examples from our nearly five million in federal research grants that have been terminated or rescinded. We will also share lessons learned from our efforts to promote visibility, advocacy, and community-driven action to support LGBTQIA+ aging research and education.

